# Effect of climate change on hematotoxicity/hepatoxicity oxidative stress, Oncorhynchus mykiss, under controlled conditions

**DOI:** 10.1371/journal.pone.0294656

**Published:** 2023-11-30

**Authors:** Gonca Alak, Fatma Betül Özgeriş, Arzu Uçar, Veysel Parlak, Esat Mahmut Kocaman, Sinan Özcan, Muhammed Atamanalp

**Affiliations:** 1 Department of Seafood Processing, Faculty of Fisheries, Atatürk University, Erzurum, Turkiye; 2 Department of Nutrition and Dietetics, Faculty of Health Sciences, Atatürk University, Erzurum, Turkiye; 3 Department of Aquaculture, Faculty of Fisheries, Atatürk University, Erzurum, Turkiye; 4 Department of Basic Sciences, Faculty of Fisheries, Atatürk University, Erzurum, Turkiye; Suez Canal University, EGYPT

## Abstract

Described as the ’main ecological factor’, temperature, strongly affects the physiological stress responses of fish. In order to evaluate the effects of temperature variations on fish culture and food value chain, the present study was designed as a climate change model. Furthermore, the present study provides a theoretical basis for a better understanding of the mechanisms of the environmentally induced changes. In this direction, we examined the blood physiology and oxidative stress responses induced by temperature variation in the rainbow trout, a temperature-sensitive cold-water fish. The obtained results showed that climate changes promoted the inhibited activities’ expressions and the development of potential tissue and hematological defense mechanisms against temperature-induced toxic damage. This study showed that climate change could be a subset of the studies on the stress physiology in aquaculture, which can be developed for new experimental designs and research collaborations. Furthermore, it highlights knowledge gaps to guide future research in this emerging field.

## 1. Introduction

In the effort to meet the food needs of a growing population, the impacts on the environment and the effects of environmental factors on food production stand out as issues that require urgent measures. Increasing demand for food, especially animal protein sources and aquaculture products, is being felt to a significant extent [1].

However, environmental changes (e.g. climate change, droughts, floods, widespread forest fires) have also led to major crop crisis. This is why food security, ending poverty and promoting sustainability are the main objectives of the UN’s 2030 Agenda for Sustainable Development Goals. Aquaculture plays an important role in achieving these goals [1–3]. Recent studies reported that increasing the consumption of seafood in diets could be a solution to meet protein requirements and support human and environmental health. Achieving these goals requires not only increasing food production, but also following best animal husbandry practices. In this context, it is important to emphasize that as a result of the growing demand for water, it has become necessary to investigate biotic and abiotic factors that may pose a threat to the industry [4–6].

Temperature is one of the key factors for the growth and survival of aquatic organisms. It is well known that temperature changes can directly affect some physiological processes, from metabolic rate, immunity and oxygen demand to contact uptake. Temperatures above the thermal threshold not only increase oxygen demand but also suppress immunity by reducing feed intake. This mechanism of action is explained by the negative impact of temperature change on aerobic metabolism. Temperature limits the solubility and availability of oxygen [7–9].

According to the Intergovernmental Panel on Climate Change, severe global warming is expected in many parts of the world by 2100 2100 [10]. In these climate change models, an increase of up to 3 °C is predicted for the next century, especially in tropical water temperatures. In addition, in aquaculture, temperature fluctuations can affect production in various dimensions, such as metabolic rate, oxygen solubility, thermal stress tolerance, tolerance to environmental pollutants, immune response, reproductive/ growth and development performance, feed intake. Overall, these negative impacts suggest that even small temperature changes can cause significant suppression of individual or population performance [8, 11–13].

However, it is known that fish actively seek their preferred temperature, which is not always possible depending on habitat characteristics and species biogeography. Consequently, experimenting with acute thermal shocks under laboratory controlled conditions is crucial to identify the basic mechanism by which fish cope with this stress. While it is known that many stress-related physiological parameters of fish are generally altered by chronic high temperature exposures, the mechanisms of these alterations are not yet fully understood [7, 12, 14, 15].

Rainbow trout (Oncorhynchus mykiss, Walbaum, 1792) is the most widely cultivated trout species in Turkey with a high commercial value [16]. Due to its fast growth and high nutritional value, it is widely cultivated in many countries. Rainbow trout live in cold, clear and well-oxygenated rivers and lakes with a water temperature of 20–21 °C [7]. When aquaculture in Turkey is analyzed, trout is the most widely cultivated species in terms of inland fish production. Trout farming covers 35.47% of our total aquaculture production in inland waters and in the sea in recent years, together with the so-called Turkish salmon, which is produced in the sea.

In recent years, aquaculture in Turkey has gained momentum in parallel with the developing technology and economic growth. As a result of overfishing and population decline, the importance of aquaculture is increasing day by day. Aquaculture studies were first started in inland waters, then they were replaced by marine environments, and with the determination and application of economic cultivation methods, the studies in the dimension of initiative have gained a sectoral structure [17].

Global warming affects water quality as well as water flow. Waters that are already heavily exposed to pollution factors will also have to absorb more pollution concentrations with the decrease in water, and with increasing water temperatures, it will negatively affect the amount of dissolved oxygen, which is very important for aquatic life, as well as many quality criteria in waters. Due to climate change and the resulting global warming, the earth’s temperature has increased by around 0.7–0.8 °C 96 over a period of approximately one hundred years in the 20th and 21st centuries. If necessary measures are not taken, temperatures will gradually increase. As a result, glaciers will gradually decrease, sea levels will rise, forest cover will decrease and natural disasters such as desertification, forest fires and extreme floods will increase. Agricultural production will be significantly affected due to drought, and many plant and animal species may face the danger of extinction due to deterioration of living conditions [18]. Mass fish mortalities are increasingly occurring in freshwater and marine habitats after unusually heat waves and hot weather. This has been described as the greatest challenge of aquatic ectothermic fauna due to the elevated frequency, intensity and duration of heat waves. The mechanism underlying all these claims is not yet fully understood. This is only possible by monitoring individual responses and physiological functions. Therefore, experimental biology is particularly important in understanding the effects of global warming on fish [19, 20]. As a result of the sensitivity of physiological parameters and to adapt to temperature changes, fish alter their endocrine processes, metabolic rates, immune and antioxidant systems [7, 9].

Based on the above strategies, in this article, we investigated the complex interaction between global warming and the physiology of freshwater aquatic organisms. In relation to the general animal physiology and oxidant levels of anthropogenic temperature increase, aquatic organisms, in particular freshwater and cold-water fish, rainbow trout (*Oncorhynchus mykiss)* are selected to study and discuss multiple biomarker responses of increasing water temperatures from a broad perspective.

## 2. Material and methods

Certified juvenile rainbow trout (*O*. *mykiss*) were obtained from Atatürk University Fisheries Faculty Inland Water Fish Application and Research Center (B.25.42.0028, 01.01.2013 by Agriculture Ministry, Turkiye) with an average weight of 20 ±0.8 g. After a 14-day acclimation period, 480 rainbow trout were treated as indicated below. The feeding was done twice a day with commercial trout pellets with the rate of 1.5% of body weight during the acclimatization periods The trail design was organized as 30 fish x 4 group with 4 replications. Fish were randomly distributed to fiberglass tanks according to the planned temperature applications. Experiment methodology, design protocol, and fish welfare were approved by Atatürk University Faculty of Veterinary Medicine, Experimental Animal Use, and Ethics Committee (approved in 28.12.2021, Acceptance No:11).

The stock ratio in these tanks (as 20 kg/ m^3^) was determined with the aim of reaching the final weight of 250±17 g. For this purpose, the limits on which water temperatures in the current working environment can be manipulated have been studied. At the determined temperatures (12±0.3°C, B: 15±0.3°C, C: 18±0.3°C, and D: 21±0.3°C), O_2_ level for fish between temperatures was adjusted by increasing the amount of water for survival comfort. Thanks to this setup, O_2_ level was kept above 7.5–8 mg L^-1^ in all groups. For reaching to 250 g final live weight day counts according to the groups were as A: 12±0.3°C (315 days), B: 15±0.3°C (300 days), C: 18±0.3°C (310 days), and D: 21±0.3°C (310 days). At the end of the time when reached to the determined weight at 4 different temperatures [A: 12±0.3°C, B: 15±0.3 °C, C:18±0.3 °C, and D: 21±0.3 °C] under controlled conditions, random samplings were made from the fish belonging to each temperature group. In order to determine the stress physiology and antioxidant status, the blood, liver and muscle tissue samples of fish (n = 15) from each tankwere taken.

### 2.1 Determination of blood physiology and antioxidant status

#### a. Determination of hematologic indexes

The rainbow trout were transferred from the tanks to an aerated clove oil anesthetic bath (60 mg/L). The samples were collected from the caudal vein with syringe. Blood samples were analyzed with PROKAN-6800VET fully automatic blood counting device. For each sample, leukocyte (WBC), hematocrit (HCT), hemoglobin (HGB), erythrocyte (RBC), mean corpuscular volume (MCV), mean corpuscular hemoglobin (MCH) and mean corpuscular hemoglobin concentration (MCHC) values were measured. The working principle of the blood counting device is based on the counting and sizing of blood cells by electrical impedance method [21]. Changes in electrical resistance produced by a particle (WBC and RBC) passing through the aperture of a sensor are measured. Since blood cells are non-conductive, the sample blood is diluted in a conductive liquid (lysis solution). A counting pool and sensing circuit counts the WBC and another counting pool and sensing circuit counts the RBC. The instrument’s microprocessor calculates and analyzes the cells (WBC and RBC) and then provides histograms. For HGB measurement, lysis is added to the blood, rapidly breaking down the red blood cell and releasing hemoglobin. Hemoglobin and lysis form a new mixture that can absorb a wavelength of 540 nm. Based on the equations, the device determines other hematological parameters (HCT, MCH and MCHC).

#### b. Determination of antioxidant enzyme activity

In order to evaluate oxidative parameters, the fish were stunned by neck crushing.

Blood samples were taken and placed in a test tube, and after 2 mL of physiological (0.9% NaCl) was added, they were centrifuged for 15 minutes in a refrigerated centrifuge at 3000 rpm at +4°C. After the formed plasma was discarded, an equal amount of saline (0.9% NaCl) was added to the remaining volume, and the erythrocytes were washed. Physiological saline added tubes were centrifuged at 2000 rpm for 8 minutes at +4 °C (this step was repeated three times). Then, after blasting the erythrocytes with cold distilled water, the obtained erythrocyte package (-20°C) was kept in deep freeze [22]. The obtained supernatants were used to determine antioxidant analyses (SOD, CAT, GPx, GSH, ROS, TOS, TAS, OSI, MPO, MDA).

*Tissue homogenate preparation*. Liver and muscle samples of fish were homogenized by adding 3 times their weight of buffer solution (KH_2_PO_4_). The tissues were thoroughly crushed with a glass baguette and homogenized for 3 minutes in an Ultrasonic Processor homogenizer. The extract was centrifuged at 9500 rpm for 30 minutes at +4 °C [23]. Clear supernatants obtained from liver and muscle tissue were used for enzyme assays (SOD, CAT, GPx, GSH, ROS, TOS, TAS, OSI, MPO, and MDA) determined in the targeted parameter supernatants. The protein concentration of each sample was determined spectrophotometrically at 595 nm according to Bradford method [24].

*Measurement of superoxide dismutase (SOD) enzyme activity*. SOD activity was determined spectrophotometer (560 nm) according to the method of NBT (nitro blue tetrazolium chloride) reduction by O-2 under light [25].

*Measurement of catalase (CAT) activity*. Catalase converts hydrogen peroxide into water and molecular oxygen with its catalytic activity. For the determination of catalase, the decreasing absorbance of hydrogen peroxide, which gives maximum absorbance at a wavelength of 240 mn, was measured [26].

*Measurement of glutathione peroxidase (GPx) activity*. GPx activity was calculated by a change in absorbance at 340 nm (decrease in values read over 3 min) with reference to Beutler [27]. GSH amount was determined via a carefully optimized enzymatic cyclic method using glutathione reductase. The sulfhydryl group of GSH reacts with DTNB (5,5’-dithio-bis2-(nitrobenzoic acid), Ellman’s reagent) to produce yellow 5-thio-2-nitrobenzoic acid (TNB). The formation of 5-thio-2-nitrobenzoic acid, which was proportional to the GSH concentration, was observed at 412 nm and 25 °C (against reactive controls).

Fish tissues’ ROS levels were obtained by modifying the Gupta et al. [28] method. The 2’,7’-dichlorofluorescein diacetate kit (DCFDA; ABCAM, DCFDA Cellular ROS Detection Assay Kit, ab113851) was used for this purpose. The sample tissues were homogenized with Tris-HCl buffer (50 mM, pH = 7.4) at a 1:10 w/v rate inside an ice bath. Homogenate samples (100 μL) of fish tissues were mixed with 1 mL of the same buffer and 5 μL of DCFDA (10 μM). The resulting mixtures were incubated for 30 min at 37°C (Lab. Companion SI-600 incubator shaker, Jelio Tech., Korea). After the incubation, the absorbance changes were measured at Ex 485 nm/Em 525 nm with a fluorescent spectrophotometer (LS55, PerkinElmer, USA).

*Total antioxidant status (TAS)*. Was measured using the method developed by Erel [29] on an auto-analyzer. In this method, while measuring the TAS level, the Fe^2+-^o-dianisidine complex forms the OH radical by forming a Fenton-type reaction with hydrogen peroxide. This strong ROS is reduced and reacts with the colorless o-dianisidine molecule at low pH and brown-yellow colored dianisidyl radicals are formed. Dianiside radicals participate in further oxidation reactions, enhancing color formation. However, the samples’ antioxidant compounds depress these oxidation-reduction reactions and diminish the color formation. TAS results were determined by reading this decrease spectrophotometrically.

Results areexpressed as mmol Trolox Equiv/L. (TOS) levels were measured with automatic colorimetric method according to [29]. In this method, the sample’ oxidants oxidize the ferrous ion-o-dianisidine complex to ferricion. The glycerol in the environment accelerates this reaction approximately three times. In acidic medium, ferric ions form a colored complex with “xylenol orange”. The intensity of this color in relation to the amount of the samples’ oxidants was measured spectrophotometrically in the autoanalyzer and TOS values were obtained. The results were recorded as μmol H_2_O_2_ Equiv/L. Oxidative stress index (OSI) was calculated as: OSI = TOS/TASx10, according to [29].

*Measurement of erythrocyte and tissue supernatant myeloperoxidase (MPO) enzyme activity*. Was performed according to Bradley et al. [30]. This method is based on the reduction of H_2_O_2_ oxidized by MPO with O-dianisidine hydrochloride and measuring the absorbance of this reduced product at 460 nm [30, 31].

*Measurement of lipid peroxidation level*. The level of malondialdehyde (MDA), which is a product of lipid peroxidation, was determined [23]. The tubes were mixed for a short time by taking 200 μl from the prepared homogenate and adding 800 μl phosphate buffer and 25 μl of a synthetic antioxidant (BHT) and 500 μl of 30% TCA. It was then incubated at -20 °C for 2 hours. After incubation, the samples were centrifuged (15 minutes 2000 rpm) and 1 ml of the supernatant was taken and transferred to another Eppendorf tube, 75 μl EDTA-Na_2_H_2_O, 250 μl TBA were added and after a light vortexing process, they were kept in a water bath (90 °C) for 15 minutes. MDA levels were calculated by reading the samples cooled to room temperature at 532 nm [23, 31].

### 2.2 Statistical analyses

At the end of the study, the data of the groups were evaluated using the SPSS (version 13) statistical package program. Duncan’s multiple comparison test was used to identify different groups following analysis of variance. The raw values from all analyzes were presented as the mean ± standard error of the groups. The statistical significance level was taken as p <0.05 in the calculations.

## 3. Results

**Fig 1:**
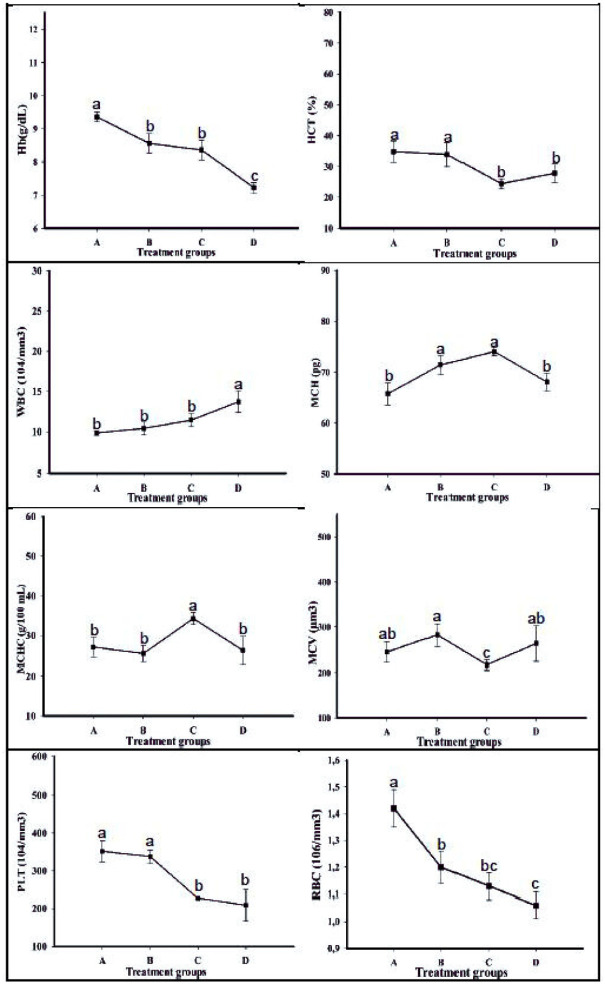
The effect of different temperature applications on O. mykiss’ hematology indexes.

It was found that different temperature applications caused alterations in rainbow trout blood samples and these changes were significant at p<0.05 level (Fig 1). RBC, Plt, Hct and Hg levels decreased due to temperature increase, while increases in WBC level were determined. RBC, Plt, Hct and Hg levels decreased by 25%, 40%, 20% and 23%, respectively, compared to the minimum value of optimum temperature group (Group A 12 °C) and WBC increased by 40% compared to the hematological index values determined in the group other than the optimum temperature values (D group 21 °C).

To understand how thermal stress affects the metabolism of this species and to assess the effect of changing temperature on blood and liver, we measured oxidative markers in these tissues, namely SOD, CAT, GPx, GSH, MDA, MPO, TOS, TAS, and OSI enzymatic activity (Table 1). The changes determined for all tissue samples were found to be important at the level of p<0.05. The optimal temperature ranges (A, B, and C) groups yielded lower MDA, MPO, and TOS values within all three tissues (blood, liver, and muscle).

**Table 1:** The effect of different temperature applications on blood, liver and muscle antioxidant status.

Tissues	Antioxidant enzyme	Treatment groups			
		A	B	C	D
Blood	SOD	1.87±0.24 ^b^	2.06±0.10 ^ab^	2.12±0.40 ^ab^	2.38±0.16 ^a^
	CAT	0.39±0.09 ^b^	2.65±0.8 ^a^	1.87±0.12 ^ab^	0.98±0.03 ^b^
	GPx	1. 07±0.08 ^a^	6. 62±0.5 ^a^	9. 67±1.03 ^a^	1. 09±0.6 ^a^
	GSH	0.52±0.01^d^	1.16±0.02 ^c^	2.88±0.27 ^b^	3.29±0.21 ^a^
	MDA	10.12±0.7 ^b^	10.37±1.04 ^b^	13.65±6.09 ^b^	26.44±6.9 ^a^
	MPO	15. 69±1.05 ^b^	22. 28±2.10 ^b^	53. 60±5.30 ^ab^	79. 25±8.3 ^a^
	TAS	0.38±0.02 ^b^	0.49±0.01 ^ab^	0.59±0.06 ^a^	0.39±0.1 ^b^
	TOS	15.34±2.3 ^b^	17.41±1.1 ^b^	24.71±1.2 ^ab^	30.66±3.1 ^a^
	OSI	40.12±2.34 ^b^	37.73±1.07 ^b^	42.09±2.63 ^b^	79.81±4.43 ^a^
Liver	SOD	1. 73±0.2 ^b^	2. 04±0.02 ^ab^	2. 12±0.1 ^ab^	2. 27±0.3 ^a^
	CAT	0.85±0.5 ^b^	0.87±0.03 ^b^	1. 67±1.4 ^ab^	3. 24±0.8 ^a^
	GPx	5. 53±0.07 ^a^	4. 96±0.1 ^a^	3. 01±0.3 ^a^	0. 52±0.02 ^a^
	GSH	0.83±0.05 ^b^	2.51±1.8 ^b^	3.13±0.5 ^b^	7.93±1.4 ^a^
	MDA	9.68±1.0 ^b^	10.18±0.9 ^b^	10.37±3.2 ^b^	30.91±5.8 ^a^
	MPO	62.00±4.5 ^b^	74.85±14.8 ^a^	20. 44±0.4 ^b^	27. 95±1.1 ^b^
	TAS	1.11±0.07 ^a^	1.06±0.08 ^ab^	1.09±0.08 ^ab^	0.92±.0.1 ^b^
	TOS	21.49±0.5 ^a^	25.06±1.8 ^a^	27.81±0.6 ^a^	43.21±1.3 ^a^
	OSI	19.58±0.5 ^b^	22.77±1.6 ^b^	25.71±0.7 ^b^	46.27±0.8 ^a^
Muscle	SOD	4. 76±0.5^b^	6. 91±0.6^ab^	13. 79±0.1^ab^	15. 93±0.5^a^
	CAT	6. 58±0.3 ^a^	10. 68±0.6 ^a^	14. 64±1.1 ^a^	16. 40±0.9 ^a^
	GPx	5. 17±0.8 ^a^	2. 58±0.3 ^a^	2. 25±0.1 ^a^	0. 22±0.1 ^a^
	GSH	0.91±0.05 ^b^	2.46±0.1 ^a^	3.04±0.4 ^a^	3.57±0.1 ^a^
	MDA	8.48±0.3 ^b^	11.19±2.5 ^b^	11.63±3.1 ^b^	24.86±1.7 ^a^
	MPO	12. 77±0.3 ^b^	30. 22±0.8 ^b^	39. 52±4.4 ^b^	96. 13±1.2 ^a^
	TAS	0.79±0.06 ^a^	0.62±0.02 ^a^	0.75±0.01 ^a^	0.78±0.02 ^a^
	TOS	10.40±0.7 ^b^	15.75±0.7 ^ab^	16.95±1.9 ^ab^	24.08±2.4 ^a^
	OSI	13.58±1.0 ^a^	30.68±2.6 ^a^	22.94±0.3 ^a^	32.14±0.9 ^a^

Lowercase superscripts (a,b,c) indicate significant differences among same colon within each experimental treatment group

Similarly, we determined the changes in muscle tissue during the possible stress process in order to evaluate the health status of fish. The findings showed changes in enzyme activities and increases in MDA and MPO levels due to temperature increase in all tissues. These changes were found to be statistically significant (p<0.05) (Table 1).

## 4. Discussion

It is well known that secondary responses to prolonged high temperature are often observed in fish, such as increased hemoglobin and hematocrit, which are thought to occur as a result of transient catecholamine fluctuations and may help fish to cope [14]. In the studies, it has been determined that epigenetic modifications can mediate and induce stress coping skills of individuals by exposing them to high temperatures in the context of global warming. These regulate secondary stress response factors that alter the distribution of essential resources (such as energy to tissues and organs, as well as compromising hydro-mineral imbalance and the immune system).

In our research findings, the change in enzyme activities revealed that seasonal variability in rainbow trout should be seriously questioned. In addition, increases in MDA levels indicate that temperature changes cause oxidative stress and acclimatization to higher temperatures leads to increased lipid oxidative damage in fish.

It is known that aquatic organisms will go through physiological difficulties, especially the decrease in oxygen will increase the metabolic demand for oxygen, as a result of global warming [32]. Dissolved oxygen and water temperature are among the most important vital factors for aquatic organisms. The relationship between these two physical factors includes thermal effects on the amount of oxygen and changes in water viscosity that inhibit the exchange of breathing gases in the gill surface layers [33]. In this study, Hb and RBC decreased significantly due to the high acclimation temperature that may result from the damage of the hematopoietic system under stress conditions. Depending on the adaptability, different species react differently at increased temperature with the different hematological responses. Changes in hematological parameters have been reported to be a common phenomenon as an answer to thermal stress (may be low or high temperature) [34, 35]. In this study, the average value of Hb and RBC was significantly reduced, which is similar to previous findings in Thai pangas and Nile tilapia [36–38]. The size, number and hemoglobin concentration of erythrocytes may be affected by changes in ambient temperature due to stressful conditions and/or insufficient oxygen supply to hematopoietic tissues [39].

On the other hand, WBC content increased significantly at higher temperature. This situation indicates the stress response of rainbow trout. As a healing response during stress exposure, high amounts of antibodies are produced and cause WBC content to increase [40–42]. Several studies are similar to our results, with some finding that the number of leukocytes in pond fish carp increases due to high temperatures [43] Previous research on Nile tilapia showed that RBC and Hb levels decreased significantly, while WBC content increased significantly at high temperature [15, 44].

Temperature directly affects the physiological process and metabolic demand of fish [45, 46]. Although fish can cope with a series of temperature changes through physiological plasticity or evolution, serious illness and death occur when they exceed their heat tolerance [47, 48]. One of the organs involved in which the oxidative state can be affected by thermal stress is the liver [49]. Many studies have found that temperature stress can induce oxidative stress in fish and activate the enzyme-induced and non-enzyme-induced antioxidant defense system [50, 51]. To maximally prevent oxidative damage caused by the production of overactive ROS, the body can upregulate its antioxidant defense system as a survival strategy to eliminate excessive ROS production caused by temperature stress [52]. ROS, which are formed in the process of aerobic respiration, are beneficial for the cell at low concentrations and serve as important signaling molecules, being induced in the dysregulation of mitochondrial function due to drastic changes in temperature. If this induction does not occur after a certain reduction, oxidative damage accumulates and cellular function declines [53, 54].

Fish are able to stabilize their body temperature in response to environmental temperature changes or migrate to areas with optimum water temperature. In aquaculture, it is important to know the effects of sub-optimal water temperatures during the transition periods from spring to summer and/or from fall to winter for fish species. A study reported that water temperature changes above optimum trigger thermal stress in fish and reduce feed intake and growth performance of fish. Thermal tolerance of fish as well as other aquatic organisms is a vital criterion in determining their resistance to temperature stress. While some studies have shown that thermal tolerance can be increased by microelements that increase oxygen holding capacity, the general view is that oxygen holding capacity does not increase thermal tolerance in fish. However, investigating how aquatic organisms adapt to the increasing water temperature, how they survive and how they resist against temperature has been one of the most important issues that scientists have emphasized. The unexpected fluctuations in water temperature that accompany climate change also cause serious losses in natural populations [55].

The general implication of temperature acclimation studies is that differences in oxidative stress are emphasized at low temperature changes rather than high temperatures [56]. Increased SOD activity due to temperature increase may be an up-regulation of ROS in these temperature treatments. SOD activity/concentration increases in the suppression of potential damage caused by this temperature change. In our research findings, the expected concordance between SOD, CAT and GPx enzyme activities was determined. Therefore, the accumulation of SOD and CAT enzymes was used to reduce the accumulation of oxidative damage by ROS, which is highly harmful. The optimum growth temperature for rainbow trout is given as 11°C in the literature [57]. Same researcher reported that temperature and flow rate changes negatively affected reproduction and growth in rainbow trout.

Similar to our results, it was found that CAT enzyme was up-regulated in temperature increases and this increase increased lipid oxidation. These results suggest that increases in SOD and CAT are effective in LPO damage in rainbow trout and differences in temperature changes strengthen the possibility of oxidative stress in fish [54].

SOD activity may have increased in both tissues to prevent oxidative stress caused by increased mitochondrial density. Increased SOD activity in both muscle and liver suggests that posttranslational modifications regulate SOD activity [58]. SOD scavenges oxygen free radicals in the body, CAT decomposes hydrogen peroxide into water, GPx reduces lipid peroxides to alcohols and tries to minimize the possibility of lipid oxidation and the damage caused by MDA to cells [9]. In our research findings, it was determined that these three enzymes cooperate to complete the antioxidant process.

The findings are supported by enzyme changes and increase in MPO and MDA that temperature causes a stress in rainbow trout. Oxidants/antioxidants can interact with each other, leading to overlapping effects [59]. From the available data, it appears that the oxidative state in the tissues and the relationship between TOS and TAS depend on temperature [60]. Regarding oxidative stress, TOS in the tissues increased at other temperatures compared to the optimal survival temperature and correlated with the MDA/MPO level of both tissues. Therefore, up-regulation of SOD in most tissues and general depression of this antioxidant enzyme suggest an increase in the level of hydrogen peroxide after thermal stress. Therefore, depression of antioxidant enzymes may be a defense strategy to help with the effectiveness of the immune system [61].

Oxidative stress participates in different cellular functions by acting as a second messenger in signal transduction. Nevertheless, they can also cause cellular damage, including oxidative damage to proteins and DNA, which creates lipid peroxidation. The thermal stress can lead to a state of oxidative stress in aquatic organisms. It has been previously observed that thermal stress can modulate the oxidative stress state in teleost fish’s various tissues [49]. The thermal stress at low temperatures has been reported in dairy fish (*Chanos chanos*) as increasing antioxidant enzymes and oxidative damage [62], while lipid peroxidation has been observed in *Piaractus mesopotamicus* under the same stressor effect [61, 63, 64]. Furthermore, lipid peroxidation may vary by species. It has been stated that carnivorous species have very low GPx activity and the highest SOD/ CAT activity in the liver, compared with herbivorous and omnivorous species, and that these differences may be effective in enzymatic antioxidant system activities [60]. This is consistent with the findings of our study, which showed that thermal stress exerts a significant oxidative effect on rainbow trout muscle and liver.

In addition, the identification of significant increases in enzyme activity with temperature changes is consistent with different studies conducted [65]. It suggests that an increase in antioxidant enzymes may protect against the harmful effects of oxidative damage caused by heat stress. Our research found that the MDA content in D in all tissues was significantly higher than in A; this indicates that oxidative stress occurs during high temperature, and the cells are damaged. The thermal stress associated with oxidative stress can vary by species and tissues, and the liver is considered a sensitive organ in determining this thermal stress [49].

## 5. Conclusion

The primary abiotic agent influencing fish physiology and growth is temperature. The effect of elevated temperatures on aquatic organisms varies depending on the severity of stress and accompanying conditions. In this study, the effect of temperature extremes on hematology and antioxidant status was discussed. In this study, we tried to present the major effects of climate change scenarios applied under controlled conditions on rainbow trout physiology. The findings have contributed to the data pools that reveal the modeling of the changes caused by each of the different climate change scenarios on parameter-based changes.

Our current research findings also confirm that rainbow trout is sensitive to increased water in terms of blood and liver oxidative stress. Therefore, the systematic study of heat stress on the qualities of rainbow trout is practical and vital. High temperature stress, with increased antioxidant enzyme activities, had a corresponding effect on the oxidative state of the fish. In addition, oxidative damage has been observed in the muscles and liver of rainbow trout, accompanied by lipid peroxidation. These findings suggest that the detection of antioxidant levels alone fails to comprehensively assess the oxidative stress state, and for the scientific and rational assessment of the oxidative stress state, it is necessary to detect TAS and TOS in addition to the calculation of OSI.

Certain fish-specific factors, along with environmental factors, plays important roles in the oxidative status of fish. Next researches on oxidative stress in fish should lead to a better understanding of fish physiology. Further studies are needed to better understand the relationship between oxidative status and environmental stressors in different fish species.
